# Use of oXiris^®^ hemoadsorption in sepsis and acute kidney injury: a retrospective cohort study in a resource-limited Colombian ICU

**DOI:** 10.3389/fneph.2025.1628181

**Published:** 2025-12-15

**Authors:** David Ballesteros, Andrea Cristina Mantilla Villarreal, Sandra Cecilia Narváez Martínez, Isabel Saravia, Susan Martínez

**Affiliations:** 1 Nephrology Service, University Hospital San José, University of Cauca, Popayán, Colombia; 2 Medical Department, Vantive, Bogotá, Colombia; 3 Epidemiology Department, EpiThinK Health Consulting, Bogotá, Colombia

**Keywords:** septic shock, acute kidney injury, renal replacement therapy, hemoperfusion, COVID-19, developing countries, hospital mortality

## Abstract

**Background:**

Septic shock with acute kidney injury (AKI) carries high mortality in resource-limited settings. The oXiris^®^ membrane enables continuous renal replacement therapy (CRRT) with endotoxin and cytokine adsorption, but data from low- and middle-income countries are scarce.

**Methods:**

We conducted a single-center retrospective cohort of adults with septic shock and KDIGO stage 2–3 AKI treated with CRRT using oXiris^®^ in a Colombian public tertiary hospital (January 2021–March 2023). The primary outcome was renal recovery, defined as dialysis independence at discharge. Secondary outcomes included in-hospital mortality, vasopressor trajectories and hemodynamics over 72 hours, intensive care unit (ICU) length of stay, and outcomes stratified by COVID-19 status.

**Results:**

Fifty patients were analyzed (median age 56.5 [IQR 46.0–66.0] years; 32% male); 21 (42%) had confirmed SARS-CoV-2 infection. Norepinephrine requirements fell from 0.303 to 0.000 µg/kg/min over 72 hours (p<0.001), and vasopressin use declined to zero (p<0.001), while mean arterial pressure increased from 74.5 to 83.0 mmHg. In-hospital mortality was 62% (31/50) and was higher in patients with greater baseline severity (APACHE II 21.5 vs 14.5 in survivors; p=0.023). ICU length of stay was 14.0 days [5.0–22.5] and was longer in survivors than non-survivors (21.0 vs 8.0 days; p<0.001). Among survivors, 63% (12/19) were dialysis-independent at discharge. COVID-19 septic shock was associated with higher crude mortality (76% vs 52%) and lower renal recovery among survivors (9.5% vs 34%) compared with non-COVID sepsis.

**Conclusions:**

In a resource-limited ICU, oXiris^®^-based CRRT was associated with rapid vasopressor de-escalation and clinically meaningful kidney recovery among survivors, but overall mortality remained high and severity-dependent. COVID-19 septic shock showed a distinct profile, with higher baseline severity, a trend toward higher mortality, and impaired renal recovery. These data support feasibility and safety of hemoadsorptive CRRT in constrained settings and justify prospective comparative evaluation.

## 1 Introduction

Sepsis and septic shock remain among the leading causes of death in critically ill adults worldwide, accounting for approximately 49 million cases and 11 million deaths annually (1). These syndromes represent a dysregulated host response to infection that triggers systemic inflammation, endothelial injury, and progressive multiorgan dysfunction (2). Although timely recognition and antimicrobial therapy have improved outcomes, the downstream inflammatory cascade continues to pose a major therapeutic challenge, particularly in settings with constrained critical care capacity. In low- and middle-income countries (LMICs), limited access to diagnostic tools, intensive care beds, and trained personnel frequently delays sepsis management, contributing to disproportionate mortality (3).

Extracorporeal blood purification has been proposed as an adjunctive intervention to mitigate the inflammatory burden in patients with sepsis-associated acute kidney injury. These techniques aim to reduce circulating concentrations of cytokines, endotoxins, and other mediators involved in organ injury. The oXiris^®^ hemofilter combines hemofiltration, cytokine adsorption, and endotoxin removal within a single membrane compatible with conventional continuous renal replacement therapy (CRRT) platforms (4, 5). The membrane consists of an AN69 copolymer modified with polyethylenimine, which enhances adsorption of negatively charged molecules, including endotoxins and proinflammatory cytokines (6). Reports from well-resourced intensive care units suggest potential benefits such as improved hemodynamic stability and reduced vasopressor requirements (7, 8). However, the feasibility, timing, and clinical impact of these therapies in LMICs remain poorly characterized.

A recent Delphi-based expert consensus developed by the Asia Pacific Sepsis Alliance emphasized the need to adapt international sepsis management guidelines to resource-limited environments (3). The panel proposed pragmatic recommendations for fluid resuscitation, vasopressor administration, and infection control that reflect the realities of LMIC healthcare systems, including reliance on clinical surrogates such as capillary refill time and urine output when advanced monitoring is unavailable. This framework underscores the importance of evaluating advanced technologies like the oXiris^®^ filter within the contextual limitations of such settings.

We conducted a retrospective cohort study of adults with sepsis or septic shock and KDIGO stage 2–3 acute kidney injury treated with continuous renal replacement therapy using the oXiris^®^ membrane. Our objective was to describe patient characteristics, timing of therapy, 72-hour hemodynamic trajectories, renal recovery at discharge, and in-hospital mortality.

## 2 Materials and methods

### 2.1 Study design and setting

We performed a retrospective observational cohort in the intensive care unit (ICU) of Hospital Universitario San José de Popayán (Popayán, Colombia) from January 1, 2021, to March 31, 2023. The ICU is a closed, multidisciplinary unit staffed by board-certified intensivists, nephrologists, critical care nurses, and respiratory therapists, with 55 ventilator-capable beds, a 1:3 nurse-to-patient ratio, bedside ultrasound, invasive hemodynamic monitoring, and CRRT capability. Intermittent shortages of consumables and staffing could affect timing and intensity of therapy (9).

The protocol was approved by the institutional ethics committee (Approval No. 47-2024). Because the data were anonymized and collected retrospectively, the requirement for informed consent was waived, in accordance with national regulations (Resolution 8430 of 1993, Colombian Ministry of Health) (10) and international ethical standards for retrospective research (11).

### 2.2 Study population

Eligible patients were adults ≥18 years admitted to the ICU with sepsis or septic shock defined per Sepsis-3 (2) and managed according to the 2021 Surviving Sepsis Campaign (SSC) recommendations (12). All included patients had acute kidney injury (AKI) KDIGO stage 2 or 3 at therapy initiation (13) and received CRRT using the oXiris^®^ hemoadsorptive membrane (Baxter, USA). Exclusions: pregnancy, age <18 years, absence of essential outcome data, and non-infectious toxic injury (paraquat poisoning) identified at data verification.

### 2.3 Standard sepsis care and protocol adherence

To contextualize outcomes and address adherence to evidence-based care, we abstracted core SSC bundle elements (12): time-to-first antibiotic dose, blood cultures before antibiotics, initial crystalloid volume in the first 3 hours, lactate measurement at presentation and re-measurement if initially elevated, timing of vasopressor initiation to target mean arterial pressure (MAP) ≥65 mmHg, and source-control timing when indicated. Where lactate or advanced monitoring were unavailable, contemporaneous documentation of capillary refill time and urine output was recorded in line with pragmatic guidance for resource-limited settings (3).

### 2.4 Timing and indications for oXiris^®^ initiation

Primary indications for initiating oXiris^®^ were sepsis or septic shock with KDIGO stage 2–3 AKI, ongoing vasopressor requirement, and clinician judgment regarding inflammatory mediator adsorption as an adjunct to CRRT. We calculated time from ICU admission to oXiris^®^ start in hours and categorized initiation as early (≤24 h) or late (>24 h) to allow exploratory comparisons requested by reviewers. The duration of oXiris^®^ circuits and total CRRT hours were recorded.

### 2.5 CRRT protocol

CRRT was delivered as continuous veno-venous hemofiltration (CVVH) on Prismaflex^®^ systems (Baxter, USA). Vascular access was ultrasound-guided (femoral or internal jugular). Standard access was a 13.5 Fr, 24-cm double-lumen catheter placed in the right internal jugular vein when feasible. Blood flow was 100–150 mL/min; effluent dose was typically 20–30 mL/kg/h, tailored to clinical targets. Anticoagulation was titrated with unfractionated heparin to a target activated partial thromboplastin time (aPTT); regional citrate with intravenous calcium was used when bleeding risk was high. Routine monitoring included arterial blood gases, electrolytes, renal indices, and lactate.

### 2.6 Data collection and variables

Data were abstracted from the electronic medical record using a standardized instrument by two independent reviewers with adjudication by consensus. Variables included:

- Demographics (age, sex) and comorbidities (diabetes, chronic heart failure, chronic kidney disease, chronic liver disease, chronic lung disease, active malignancy, immunosuppression).
- Illness severity: SOFA and APACHE II scores calculated at or within 6 hours of oXiris^®^ initiation; when a component was missing, the score was marked not evaluable to avoid imputation.
- Sepsis source and etiology (respiratory, intra-abdominal, urinary, skin/soft tissue, bloodstream; culture or PCR results when available).
- Hemodynamics and perfusion: MAP and norepinephrine/vasopressin doses at baseline (day 0) and days 1–3; lactate at the same time points.
- CRRT parameters: modality, dose, circuit count/duration, anticoagulation strategy, total CRRT hours; post-oXiris^®^ intermittent hemodialysis.
- Process-of-care: SSC bundle items as above (12).
- Virology: confirmed SARS-CoV-2 infection status.

Missing data were quantified and reported; no statistical imputation was performed.

### 2.7 Outcomes

The primary outcome was renal recovery defined as dialysis independence at hospital discharge. Secondary outcomes were in-hospital mortality, time to death, time to renal recovery, trends in vasopressor requirements (days 0–3), MAP and lactate trajectories, and need for intermittent hemodialysis after oXiris^®^.

### 2.8 Statistical analysis

Analyses used R 4.3.3 (14). Continuous variables were checked for normality by inspection and Kolmogorov–Smirnov testing; they are presented as mean ± SD or median [IQR] as appropriate. Categorical variables are frequencies and percentages. Survivor vs non-survivor comparisons used Wilcoxon rank-sum (continuous) and Fisher’s exact (categorical) tests. Repeated measures of vasopressors, MAP, and lactate over days 0–3 used the Friedman test. Kaplan–Meier methods were applied to survival and renal recovery with censoring at ICU discharge for survivors and at discharge for those without recovery, respectively.

Subgroup analyses compared COVID-19–positive versus non-COVID septic shock across baseline variables, hemodynamic evolution, and outcomes. Continuous variables were tested with Wilcoxon rank-sum; categorical variables with Fisher’s exact. Within-group vasopressor trajectories were assessed using Friedman tests for repeated measures on complete cases. ICU length of stay was analyzed as a continuous variable and compared between survivor groups using Wilcoxon rank-sum; correlation with APACHE II used Spearman’s ρ. Missing data were analyzed by complete-case approach without imputation. All tests were two-sided with p < 0.05 considered statistically significant.

## 3 Results

### 3.1 Study population and participant flow

**Figure 1:**
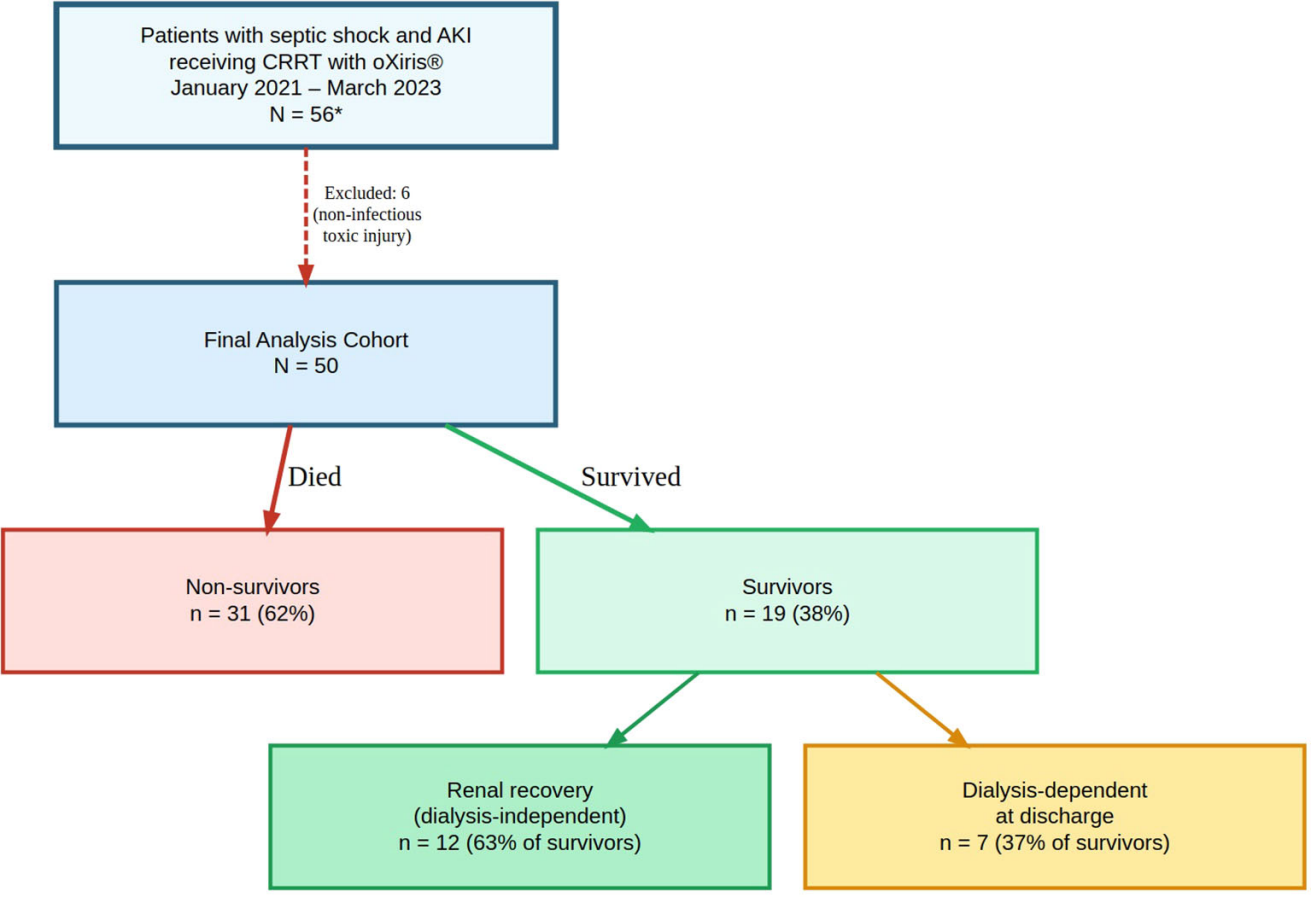
Study cohort assembly and outcomes (N = 50). Adult patients with septic shock and KDIGO stage 2–3 acute kidney injury who received continuous renal replacement therapy using the oXiris® membrane between January 2021 and March 2023 were screened. *Six patients with non-infectious toxic ingestion (paraquat) were excluded, yielding a final analysis cohort of 50. Final in-hospital outcomes: 31 deaths (62%) and 19 survivors (38%).

Between January 1–2021 and March 31 2023, 50 adults with sepsis or septic shock and KDIGO stage 2–3 acute kidney injury received CRRT with the oXiris^®^ hemoadsorptive membrane and were included in the analysis after exclusion of non-infectious toxic ingestions. Overall in-hospital mortality was 62% (31/50); 38% (19/50) survived to discharge (**Figure 1**).

### 3.2 Baseline demographic and clinical characteristics

Baseline characteristics are summarized in **Table 1**. Median age was 56.5 years [IQR 46.0–66.0]; 32% were male. Non-survivors had higher APACHE II scores than survivors (21.5 [16.0–27.0] vs 14.5 [7.0–23.5]; p=0.023).

**Table 1:** Baseline demographic, clinical, and disease-severity characteristics of critically ill adults with septic shock and acute kidney injury treated with oXiris^®^ hemoadsorption.

Characteristic	Overall (N = 50)	Non-survivors (n=31)	Survivors (n=19)	P-value
Demographics				
Age (years), median [IQR]	56.5 [44.0, 66.0]	57.0 [49.0, 68.0]	50.0 [36.0, 63.0]	0.123
Sex, n (%)				0.538
Female	34 (68%)	19 (61%)	15 (79%)	
Male	16 (32%)	12 (39%)	4 (21%)	
Illness severity				
APACHE II score†, median [IQR]	20.0 [11.0–26.0]	21.5 [16.0–27.0]	14.5 [7.0–23.5]	0.023
Charlson Comorbidity Index‡, n (%)				0.530
0	31 (62%)	17 (55%)	14 (74%)	
1	17 (34%)	12 (39%)	5 (26%)	
2	2 (4.0%)	2 (6.5%)	0 (0%)	
Comorbidities, n (%)				
Diabetes mellitus	15 (30%)	9 (29%)	6 (32%)	>0.999
Hypertension	16 (32%)	12 (39%)	4 (21%)	0.216
Obesity	11 (22%)	8 (26%)	3 (16%)	0.489
Chronic heart failure	1 (2.0%)	1 (3.2%)	0 (0%)	>0.999
Chronic obstructive pulmonary disease	1 (2.0%)	1 (3.2%)	0 (0%)	>0.999
Active malignancy	2 (4.0%)	2 (6.5%)	0 (0%)	0.510
Immunosuppression	1 (2.0%)	1 (3.2%)	0 (0%)	>0.999
Source of infection, n (%)				0.076
Respiratory	25 (50%)	19 (61%)	6 (32%)	
Intra-abdominal	16 (32%)	7 (23%)	9 (47%)	
Skin/soft tissue	3 (6.0%)	2 (6.5%)	1 (5.3%)	
Undocumented	6 (12%)	3 (9.7%)	3 (16%)	
Clinical context				
Post-operative cardiovascular surgery, n (%)	1 (2.0%)	0 (0%)	1 (5.3%)	0.400
SARS-CoV-2 confirmed, n (%)	21 (42%)	16 (52%)	5 (26%)	0.079
Baseline laboratory values				
Serum creatinine (mg/dL), median [IQR]	3.45 [2.44–4.09]	3.44 [2.44–3.98]	3.45 [2.19–4.53]	0.905
Lactate dehydrogenase (U/L), median [IQR]	588.0 [346.0–924.0]	704.0 [376.0–1,511.0]	456.5 [271.0–593.0]	0.017
Ferritin (ng/mL), median [IQR]§	1,424.0 [874.0–2,186.0]	1,528.0 [945.0–2,186.0]	913.0 [696.0–2,181.0]	0.368
D-dimer (ng/mL), median [IQR]§	1,044.7 [766.45–2,863.0]	1,009.25 [616.0–3,433.0]	1,044.7 [895.45–1,673.0]	0.977
CRRT characteristics				
oXiris^®^ duration (days), n (%)				0.707
1	11 (22%)	8 (26%)	3 (16%)	
2	10 (20%)	5 (16%)	5 (26%)	
3	23 (46%)	14 (45%)	9 (47%)	
4	4 (8.0%)	2 (6.5%)	2 (11%)	
5	1 (2.0%)	1 (3.2%)	0 (0%)	
6	1 (2.0%)	0 (0%)	1 (5.3%)	
Total CRRT hours, median [IQR]	72.0 [30.0–72.0]	61.5 [27.0–72.0]	72.0 [35.0–72.0]	0.449
Number of oXiris^®^ circuits, n (%)				>0.999
1	38 (76%)	23 (74%)	15 (79%)	
2	12 (24%)	8 (26%)	4 (21%)	
Intermittent HD post-oXiris^®^, n (%)	29 (58%)	12 (39%)	17 (89%)	´0.003
ICU and renal outcomes				
ICU length of stay (days), median [IQR]	14.0 [5.0–22.5]	8.0 [4.0–16.0]	21.0 [14.5–27.5]	<0.001
Dialysis-independent at discharge, n (%)††	12 (24%)	—	12 (63% of survivors)	—
Dialysis-dependent at discharge, n (%)††	7 (14%)	—	7 (37% of survivors)	—

† APACHE II scores calculated within 24 hours of ICU admission.

‡ Charlson Comorbidity Index calculated from available comorbidities.

§ Ferritin and D-dimer available in a subset because of incomplete records.

†† Dialysis status at discharge was assessed only among survivors (n=19).

ICU length of stay was defined as the interval in days between ICU admission and ICU discharge.

Statistical tests: Wilcoxon rank-sum for continuous variables; Fisher exact for categorical variables.

Data are median [IQR] or n (%). Missing values were not imputed. Serial lactate and full Sequential Organ Failure Assessment data were not consistently available in the source record.

Respiratory infections were the leading source (50%), followed by intra-abdominal infection (32%), skin and soft tissue infection (6%), and undocumented source (12%). SARS-CoV-2 infection was confirmed in 21 patients (42%) and was more common in non-survivors than survivors (52% vs 26%; p=0.079). Lactate dehydrogenase was higher in non-survivors (704 [376.0–1,511.0] U/L) than survivors (456.5 [271.0–593.0] U/L; p=0.017). Other laboratory values, including creatinine, ferritin, and D-dimer, did not differ significantly.

### 3.3 ICU length of stay

Median ICU length of stay was 14.0 days [IQR 5.0–22.5], ranging from 1.0 to 54.0 days (**Table 1**). Survivors remained longer than non-survivors (21.0 [14.5–27.5] vs 8.0 [4.0–16.0] days; p = 0.000928), reflecting the time required for organ recovery, ventilator weaning, and dialysis liberation in those who ultimately survived. No correlation was seen between ICU stay and baseline illness severity as measured by APACHE II score (ρ = –0.133; p = 0.356), indicating that initial physiologic derangement alone did not predict downstream resource use.

### 3.4 Illness severity and infection characteristics

Non-survivors exhibited higher baseline severity, with median APACHE II 21.5 [16.0–27.0] compared to 14.5 [7.0–23.5] in survivors (p=0.023). LDH was likewise elevated among non-survivors (704 vs 456.5 U/L, p=0.017). The infection pattern mirrored the COVID-19 era: respiratory foci carried the highest mortality (76%), while intra-abdominal infections demonstrated lower mortality (44%).

### 3.5 Hemodynamic response and vasopressor requirements

**Figure 2:**
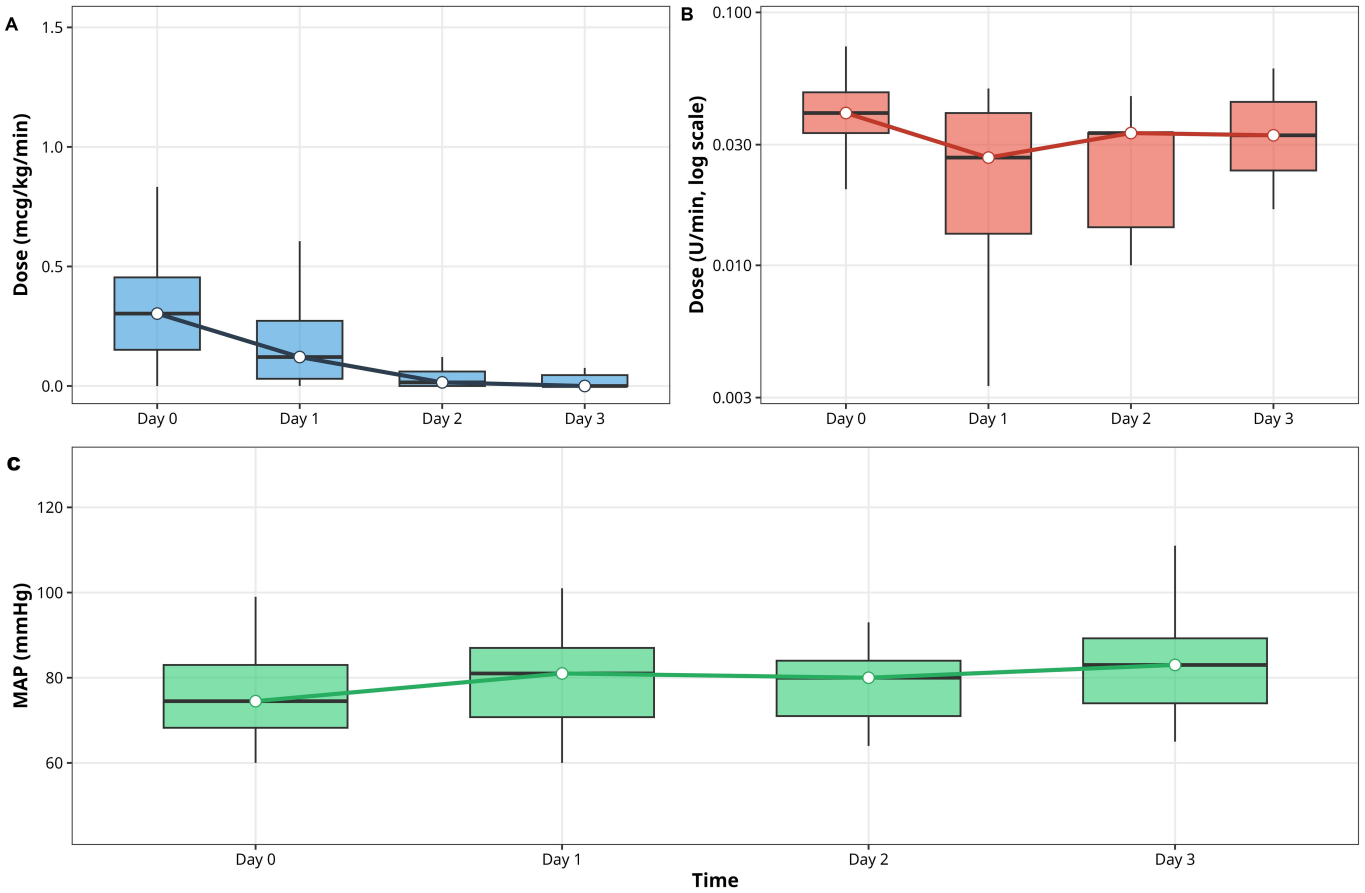
Hemodynamic trajectories during oXiris® therapy (0–72 h). **(A)** Norepinephrine (mcg/kg/min). **(B)** Vasopressin (U/min). Medians ± IQR; Friedman p values reported in **Table 2**. **(C)**: Mean arterial pressure (MAP)(mmHg).

Vasopressor trajectories are presented in **Table 2** and **Figure 2**.

**Table 2:** Temporal evolution of vasopressor requirements and mean arterial pressure during oXiris^®^ therapy.

Parameter	Day 0 (Baseline)	Day 1	Day 2	Day 3	P-value†
Norepinephrine (mcg/kg/min)					
Median [IQR]	0.303 [0.151–0.454]	0.121 [0.030–0.273]	0.015 [0.000–0.061]	0.000 [0.000–0.045]	<0.001
n with data	49	49	49	49	
Vasopressin (U/min)					
Median [IQR]	0.018 [0.000–0.040]	0.000 [0.000–0.017]	0.000 [0.000–0.000]	0.000 [0.000–0.000]	<0.001
n with data	50	50	50	50	
Mean arterial pressure (mmHg)					
Median [IQR]	74.5 [68.25–83.0]	81.0 [70.75–87.0]	80.0 [71.0–84.0]	83.0 [74.0–89.25]	0.151
*n with data*	28	28	28	28	

Data are median [interquartile range]. †Friedman test for repeated measures using complete cases only. Analysis restricted to patients with complete data across all four time points to ensure valid repeated measures comparison.

Serum lactate concentrations were not available in the clinical database and thus could not be analyzed. Lactate clearance, a key metric of resuscitation adequacy and metabolic recovery, remains an important unmeasured outcome in this cohort.

Norepinephrine requirements decreased from 0.303 µg/kg/min [IQR 0.151–0.454] at baseline to 0.000 [IQR 0.000–0.045] by 72 h (p<0.001, Friedman test).

Vasopressin dosage declined from 0.018 U/min [IQR 0.000–0.040] to 0.000 [IQR 0.000–0.000] (p<0.001).

Concurrently, mean arterial pressure increased from 74.5 to 83.0 mmHg (p=0.151).

Because serum lactate data were unavailable, metabolic correlation of perfusion improvement could not be confirmed.

### 3.6 Renal function outcomes

Among the 19 survivors, 12 (63%) were dialysis-independent at hospital discharge and 7 (37%) remained dialysis-dependent. Overall renal recovery at discharge, defined as dialysis independence, was therefore observed in 12 of 50 patients (24%) and occurred almost exclusively among survivors.

### 3.7 Survival outcomes

**Figure 3:**
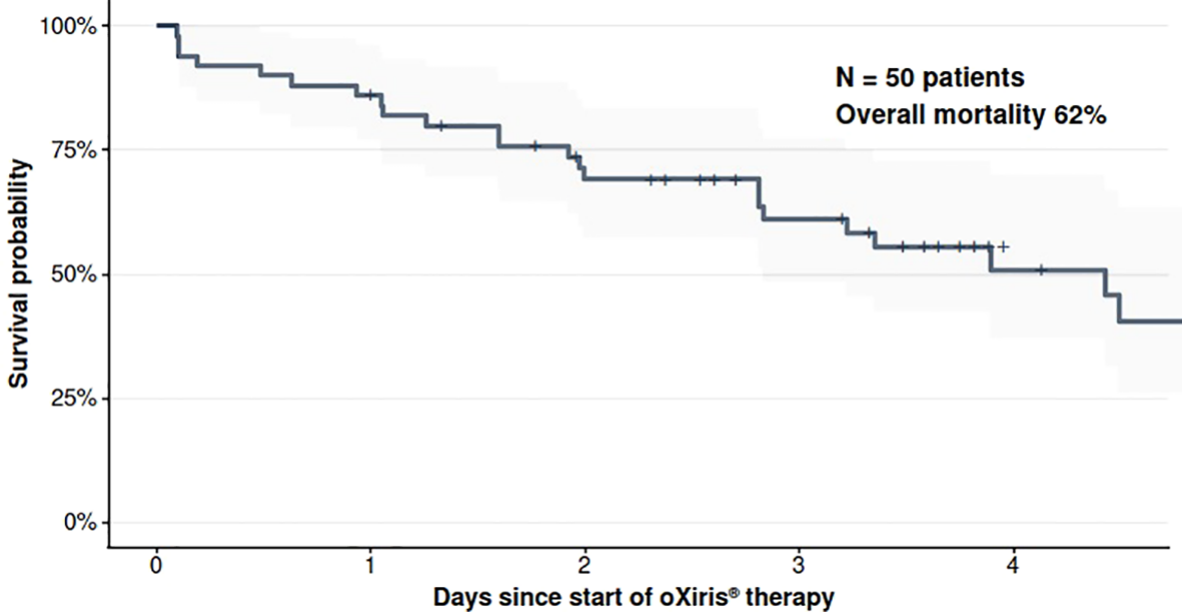
Kaplan-Meier curve for overall in-hospital survival. Survival probability from initiation of oXiris® therapy through hospital discharge or death. The curve represents the entire cohort (N = 50). Shaded area represents the 95% confidence interval.

Overall in-hospital mortality was 62% (31/50 patients). Kaplan–Meier analysis using therapy duration as the time metric demonstrated progressive decline in survival throughout the treatment period (**Figure 3**).

### 3.8 Subgroup analysis: COVID-19 versus non-COVID septic shock

Patients with laboratory-confirmed SARS-CoV-2 infection (n = 21, 42%) were compared with patients without COVID-19 (n = 29, 58%) to assess phenotypic differences and clinical course (**Table 3**). COVID-19 cases were older (57.0 vs 55.0 years; p = 0.215) and presented with greater acute severity. Median APACHE II was 25.0 [16.0–28.0] in COVID-19 versus 17.0 [9.0–23.0] in non-COVID sepsis (p = 0.075). Respiratory infection was the dominant source in COVID-19 (95% vs 17%; p < 0.001), whereas non-COVID patients more often had intra-abdominal infection or other septic sources. Ferritin levels were markedly higher in COVID-19 (1–528 vs 269 ng/mL; p = 0.002), consistent with intensified inflammatory burden.

**Table 3:** Baseline demographic, clinical, and disease-severity characteristics of critically ill adults with septic shock and acute kidney injury treated with oXiris^®^ hemoadsorption.

Characteristic	Overall (N = 50)	Non-COVID (n=29)	COVID-19 (n=21)	P-value
Age (years), median [IQR]	56.5 [44.0, 66.0]	55.0 [37.0, 66.0]	57.0 [49.0, 68.0]	0.215
Sex, n (%)				0.075
Female	33 (66%)	16 (55%)	17 (81%)	
Male	17 (34%)	13 (45%)	4 (19%)	
APACHE II score, median [IQR]	20.0 [9.0, 26.0]	17.0 [9.0, 23.0]	25.0 [16.0, 28.0]	0.075
Charlson Comorbidity Index, n (%)				0.308
0	31 (62%)	19 (66%)	12 (57%)	
1	17 (34%)	10 (34%)	7 (33%)	
2	2 (4.0%)	0 (0%)	2 (9.5%)	
Source of infection, n (%)				<0.001
Respiratory	25 (50%)	5 (17%)	20 (95%)	
Intra-abdominal	16 (32%)	15 (52%)	1 (4.8%)	
Skin/soft tissue	3 (6.0%)	3 (10%)	0 (0%)	
Undocumented / other	6 (12%)	6 (21%)	0 (0%)	
Creatinine (mg/dL), median [IQR]	3.47 [2.44, 4.28]	3.23 [1.79, 4.09]	3.52 [3.22, 4.28]	0.185
Lactate dehydrogenase (U/L), median [IQR]	588.0 [346.0, 924.0]	559.5 [239.5, 1,477.5]	615.0 [376.0, 745.0]	0.759
Ferritin (ng/mL), median [IQR]	1,407.5 [785.0, 2,183.5]	269.0 [166.0, 348.0]	1,528.0 [914.0, 2,186.0]	0.002
D-dimer (ng/mL), median [IQR]	1,044.7 [766.45, 2,863.0]	1,074.4 [985.0, 3,015.0]	936.5 [764.9, 1,727.0]	0.619
Baseline MAP (mmHg), median [IQR]	73.0 [66.0, 83.0]	73.0 [65.0, 81.5]	77.0 [70.0, 85.0]	0.199
Baseline norepinephrine (µg/kg/min), median [IQR]	0.303 [0.091, 0.439]	0.363 [0.182, 0.485]	0.227 [0.000, 0.333]	0.0258
Vasopressin use at baseline, n (%)	26 (52%)	17 (59%)	9 (43%)	0.390
oXiris^®^ duration (days), n (%)				0.769
1	11 (22%)	7 (24%)	4 (19%)	
2	9 (18%)	6 (21%)	3 (14%)	
3	23 (46%)	13 (45%)	10 (48%)	
4	4 (8.0%)	1 (3.4%)	3 (14%)	
5	2 (4.0%)	1 (3.4%)	1 (4.8%)	
6	1 (2.0%)	1 (3.4%)	0 (0%)	
Total CRRT hours, median [IQR]	72.0 [33.0, 72.0]	72.0 [33.0, 72.0]	72.0 [37.0, 72.0]	0.355
Number of oXiris^®^ circuits, n (%)				0.849
1	37 (74%)	22 (76%)	15 (71%)	
2	12 (24%)	6 (21%)	6 (29%)	
≥3	1 (2.0%)	1 (3.4%)	0 (0%)	
Post-oXiris^®^ intermittent HD, n (%)	28 (56%)	17 (59%)	11 (52%)	0.775
Norepinephrine at 72 h (µg/kg/min), median [IQR]	0.000 [0.000, 0.045]	0.000 [0.000, 0.015]	0.030 [0.000, 0.076]	0.102
Absolute norepinephrine reduction (µg/kg/min)†	0.18 [0.08, 0.36]	0.30 [0.15, 0.45]	0.12 [0.00, 0.23]	0.003
Norepinephrine reduction (%), median [IQR]	100.00 [83.33, 100.00]	100.00 [90.00, 100.00]	90.91 [55.00, 100.00]	0.087
In-hospital death, n (%)	31 (62%)	15 (52%)	16 (76%)	0.139
Renal recovery at discharge, n (%)‡	12 (24%)	10 (34%)	2 (9.5%)	0.051
ICU length of stay (days), median [IQR]	14.0 [5.0, 23.0]	16.0 [5.0, 27.0]	13.0 [5.0, 18.0]	0.798

† “Absolute norepinephrine reduction” refers to the change in norepinephrine infusion rate from baseline to hour 72.

‡ Renal recovery and dialysis dependence at discharge were evaluated among survivors only (n=19).

COVID-19 status was defined as laboratory-confirmed SARS-CoV-2 infection.

ICU length of stay was defined as the interval in days between ICU admission and ICU discharge.

Statistical tests: Wilcoxon rank-sum (continuous variables); Fisher’s exact (categorical variables).

Data are median [IQR] or n (%). Analyses are complete-case.

Both strata demonstrated significant norepinephrine de-escalation during the first 72 hours of oXiris^®^-based CRRT (Friedman p = 0.000651 in COVID-19; p = 1.29×10^-^¹¹ in non-COVID). Baseline norepinephrine dose was higher in non-COVID patients (0.363 vs 0.227 µg/kg/min; p = 0.0258), but by hour 72 residual norepinephrine was similar between groups (0.000 vs 0.030 µg/kg/min; p = 0.102). Absolute norepinephrine reduction from baseline to 72 hours was greater in the non-COVID group (0.30 [0.15–0.45] vs 0.12 [0.00–0.23] µg/kg/min; p = 0.003), indicating more complete vasopressor liberation.

In-hospital mortality was 76% (16/21) in COVID-19 versus 52% (15/29) in non-COVID sepsis (Fisher p = 0.139; odds ratio 0.34 [95% CI 0.08–1.33]). Among survivors, renal recovery at discharge was less frequent in COVID-19 (9.5% vs 34%; p = 0.051). ICU stay duration was comparable between strata (13.0 [5.0–18.0] vs 16.0 [5.0–27.0] days; p = 0.798), suggesting that once patients progressed to refractory septic shock requiring hemoadsorptive CRRT, total ICU resource use was similar across etiologies.

### 3.9 Exploratory subgroup analyses

#### 3.9.1 Mortality by illness severity

When stratified by APACHE II score tertiles, mortality increased with higher baseline severity:

- Low tertile (APACHE II 4–15, n=19): 42% mortality (8/19).
- Medium tertile (16–25, n=18): 66% mortality (12/18).
- High tertile (26–38, n=13): 85% mortality (11/13).

**Figure 4:**
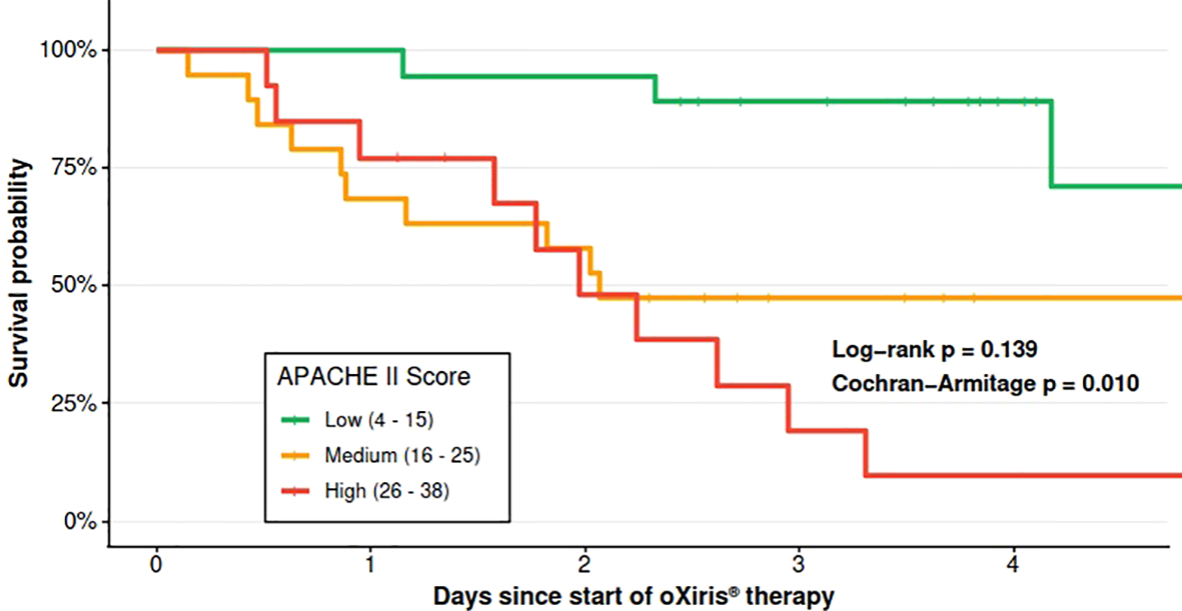
Survival by APACHE II tertiles. Kaplan-Meier curves stratified by baseline illness severity.

Kaplan–Meier curves showed clear separation consistent with this gradient (**Figure 4**). The Cochran–Armitage trend test was p=0.010, and the log-rank test was p=0.139.

#### 3.9.2 Mortality by infection source

**Figure 5:**
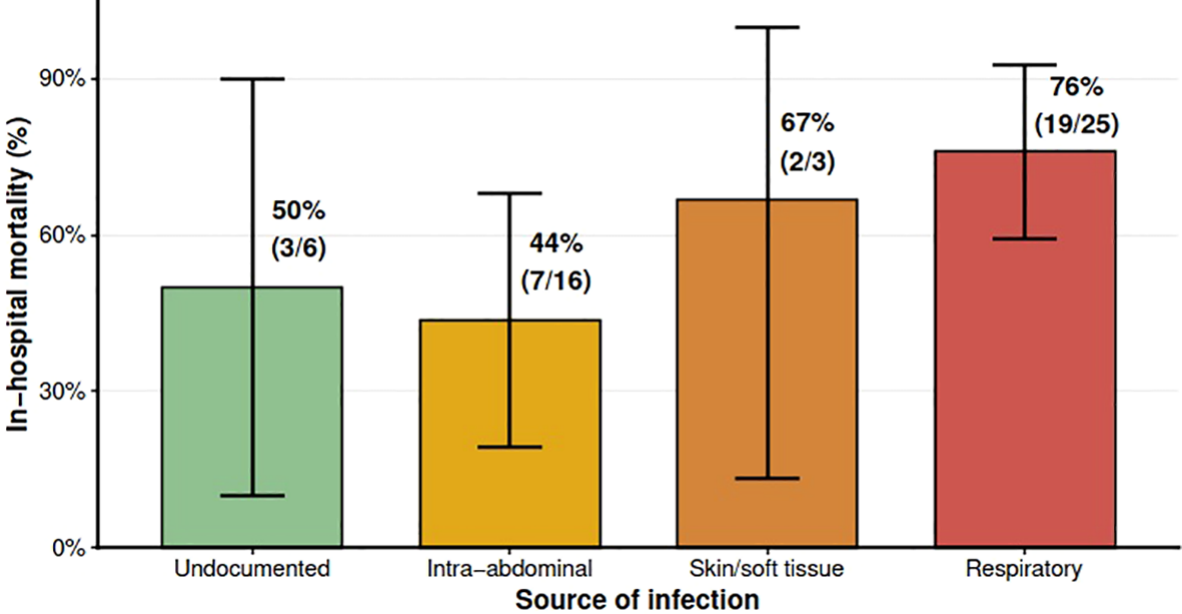
In-hospital mortality by infection source. Bar chart with mortality % ± 95% CI and n/N labels.

Mortality differed across infection types (Fisher p=0.138): respiratory 76% (19/25), skin/soft tissue 67% (2/3), intra-abdominal 44% (7/16), and undocumented 33% (2/6) (**Figure 5**).

### 3.10 Summary of clinical outcomes

Of the 50 patients, 31 (62%) died during hospitalization. Among the 19 survivors, 12 (63%) were discharged dialysis-independent and 7 (37%) remained dialysis-dependent. No serious adverse events were reported, and no complications were attributed to the hemoadsorptive circuits or to vascular access.

### 3.11 Data completeness and methodological limitations

This was a single-center study.

Baseline SOFA scores, serum lactate, and timing variables (hospital-to-ICU, ICU-to-oXiris^®^ intervals) were unavailable and could not be retrospectively obtained within this revision window.

These missing parameters limit comparison with other sepsis cohorts and preclude full assessment of metabolic resuscitation or Surviving Sepsis Campaign bundle adherence.

Interleukin-6 measurements and endotoxin activity assays were not available.

All available variables are reported with explicit denominators in **Tables 1** and **2**.

## 4 Discussion

This retrospective cohort study characterized critically ill adults with septic shock and acute kidney injury treated with the oXiris^®^ hemoadsorptive membrane in a resource-limited Colombian public hospital. We observed rapid vasopressor de-escalation during the first 72 hours of therapy, with norepinephrine decreasing from 0.303 to 0.000 µg/kg/min (p<0.001) and vasopressin falling to 0.000 U/min (p<0.001), while mean arterial pressure increased from 74.5 to 83.0 mmHg (p=0.151). Overall in-hospital mortality was 62% (31/50) and tracked with baseline severity, as shown by higher APACHE II scores in non-survivors than survivors (21.5 vs 14.5; p=0.023). Among survivors, 63% (12/19) were discharged dialysis-independent.

These findings add real-world data from a Latin American setting where extracorporeal blood purification is rarely reported. While causal inference is not possible without a control group, the physiological pattern, rapid de-escalation of vasopressors with stable macrohemodynamics, supports the feasibility of implementing advanced renal support in constrained environments.

The hemodynamic trends observed in our cohort parallel those reported in well-resourced ICUs. Schwindenhammer et al. documented an 88% norepinephrine reduction and improved predicted vs. observed mortality in French septic shock patients treated with oXiris^®^ (5). Similarly, Lumlertgul and Srisawat found significant vasopressor decreases (−45.9%, p=0.02) and cardiovascular SOFA improvement in Thai patients (15). In a randomized crossover trial, Broman et al. demonstrated significant removal of endotoxin and inflammatory mediators (TNF-α, IL-6, IL-8, IFN-γ) alongside reduced lactate concentrations compared with conventional filters (16).

The median ICU stay of 14.0 days in this cohort reflects the intensity of care required to support refractory septic shock with hemoadsorptive CRRT in a constrained public system. Survivors remained in the ICU for a median of 21.0 days, compared with 8.0 days in non-survivors, indicating that fatal cases tended to progress to death early, while survivors required prolonged organ support, ventilator weaning, and rehabilitation. ICU stay showed no meaningful relationship with baseline APACHE II score (ρ = –0.133; p = 0.356), suggesting that initial physiologic derangement did not predict duration of critical care needs. For a 50-patient series, this level of bed occupancy represents a substantial opportunity cost in a Colombian public ICU operating at or near capacity.

These studies typically initiate therapy early (≤24 hours after shock onset) and under continuous hemodynamic monitoring. In contrast, delayed initiation and intermittent monitoring characterized our setting due to staffing ratios (1:2) and supply interruptions. Despite these limitations, the magnitude of vasopressor reduction exceeded that in some structured trials (17–19), indicating preserved physiological responsiveness despite operational constraints.

The absence of lactate clearance data in our study limits interpretation of tissue perfusion recovery. Lactate kinetics remain a key marker of resuscitation adequacy and prognosis (2, 12). Without these data, improved MAP cannot be equated with restored microcirculatory flow, a critical gap for future prospective research.

The in-hospital mortality of 62% in this cohort is consistent with reports from low- and middle-income countries, where septic shock mortality often ranges from 50% to 70% (3, 9, 20). These disparities stem from systemic limitations: delayed recognition, scarce antimicrobials, limited organ support, and high nurse-to-patient ratios (3, 20).

Severity at presentation remained the main determinant of outcome. When patients were stratified by APACHE II tertiles at the time oXiris^®^ therapy was initiated, mortality rose stepwise with increasing baseline severity, and the survival curves separated accordingly (**Figure 4**). The Cochran–Armitage test confirmed a graded increase in mortality across APACHE II strata (p=0.010), and the log-rank comparison of Kaplan–Meier curves showed persistent separation over time (p=0.139). This pattern echoes experiences from other resource-limited intensive care units, including checklist-based sepsis programs that improved processes of care but did not uniformly change survival (9). Together, these studies highlight that mortality reduction in LMICs depends primarily on strengthening fundamental sepsis workflows rather than isolated technological interventions.

The implementation of oXiris^®^ in this public hospital underscores both feasibility and systemic fragility. As described by Srisawat and Chakravarthi (21), LMICs face persistent obstacles: shortages of trained staff, financial constraints, and unpredictable supply chains. Our unit experienced similar barriers, including stockouts and incomplete lactate monitoring.

The clinical profile of renal outcomes was consistent with published experience. Among survivors, 63% (12/19) left the hospital without dialysis, and the remaining patients were discharged on intermittent hemodialysis. The high rate of post-oXiris^®^ intermittent hemodialysis among survivors reflects survival bias: patients must live long enough to transition to intermittent clearance. Limited premorbid data prevent us from distinguishing full renal recovery from partial stabilization at discharge.

During the pandemic period, 42% of patients had confirmed SARS-CoV-2 infection, predominantly among non-survivors. Respiratory infections carried the highest mortality (76%), similar to rates in COVID-19-related septic shock reported by Villa et al. (19) and Borbolla-Flores et al. (22). Intra-abdominal infections showed lower mortality (44%), which likely reflects the benefit of timely source control (12). These patterns underscore how infection source and access to surgical intervention shape outcomes independently of adjunctive extracorporeal support.

COVID-19 septic shock showed a biologically distinct profile compared with non-COVID sepsis. COVID-19 cases were characterized by predominantly respiratory infection, higher ferritin, and higher illness severity at presentation. Mortality reached 76% in COVID-19 versus 52% in non-COVID sepsis, and renal recovery among survivors was lower in COVID-19 (9.5% vs 34%). These patterns are consistent with coronavirus disease 2019–associated multiorgan injury, including endothelial dysfunction and tubular damage, described in prior critical care series of COVID-19 with renal failure.

Both strata demonstrated rapid vasopressor de-escalation with hemoadsorptive CRRT, supporting a shared cytokine-driven vasoplegic physiology. At the same time, the absolute norepinephrine reduction over 72 hours was greater in non-COVID sepsis, and COVID-19 patients retained measurable vasopressor requirements at 72 hours despite CRRT. The comparable ICU length of stay between groups, despite different outcomes, suggests that once shock severity required oXiris^®^-based CRRT, total resource utilization was similar regardless of etiology.

This study provides one of the first detailed accounts of oXiris^®^ implementation in a Latin American public ICU. Strengths include comprehensive hemodynamic tracking, validated APACHE II-based severity stratification, and transparent reporting of data completeness. The description of local operational context, staffing, supply reliability, and monitoring limitations, enhances interpretability for similar institutions and aligns with recent calls for context-aware sepsis research (3, 9, 20).

Limitations mirror those of retrospective work in resource-limited settings. SOFA scores could not be reconstructed because of missing PaO_2_/FiO_2_, Glasgow Coma Scale, and coagulation data; serial lactate was unavailable; and process-of-care timestamps were incomplete. The absence of a control group limits causal inference, and the sample size (n=50) restricts power for adjusted modeling. Selection bias is possible because therapy initiation depended on clinician judgment and consumable availability; variability in circuit duration and timing adds heterogeneity. Despite these constraints, the internal consistency of the physiological signal and the severity-dependent mortality support the credibility of the observations.

The COVID-19 subgroup analysis was exploratory and underpowered (21 COVID-19 vs 29 non-COVID patients). Observed mortality differences (76% vs 52%; Fisher p = 0.139) and renal recovery differences (9.5% vs 34%; p = 0.051) should be interpreted cautiously. The analysis is also subject to confounding by indication: oXiris^®^ initiation was clinician-driven during pandemic surges when illness severity and resource pressure were both high. Missing data limited physiologic profiling; serial lactate and complete Sequential Organ Failure Assessment elements were not consistently available, and sepsis bundle timing (time to antibiotics, source control) could not be reconstructed retrospectively.

For clinicians in low-resource ICUs, these findings indicate that oXiris^®^ can be operationalized safely within existing CRRT programs. Yet, its routine use should not precede optimization of foundational sepsis care: timely antibiotics, source control, adequate staffing, and reliable monitoring (12).

Future research priorities include pragmatic multicenter trials in LMICs to compare oXiris^®^ versus standard membranes under real-world conditions, implementation-science studies to identify adoption barriers, and economic analyses to assess cost-effectiveness. Regional registries could generate robust observational data to inform local guidelines. Mechanistic studies evaluating cytokine clearance and hemodynamic biomarkers will clarify whether physiological responses translate into improved survival.

## 5 Conclusion

In this real-world cohort of adults with septic shock and severe AKI managed in a resource-limited Colombian ICU, oXiris^®^-based CRRT was feasible and safe. Vasopressor needs fell over the first 72 hours, and most survivors achieved dialysis liberation by discharge.

Survivors required prolonged ICU care (median 21 days), underscoring the resource implications of supporting recovery in this setting, while non-survivors tended to die early in the ICU.

COVID-19 septic shock displayed higher baseline severity, persistent vasopressor exposure, lower renal recovery, and a higher crude mortality rate than non-COVID sepsis, despite a broadly similar hemodynamic response to hemoadsorptive CRRT.

Although causality cannot be inferred in the absence of a control group, these findings indicate that hemoadsorptive CRRT can be delivered in low-resource ICUs and warrant prospective comparative evaluation alongside strengthened sepsis care processes.

## Data Availability

The raw data supporting the conclusions of this article will be made available by the authors, without undue reservation.
